# T lymphocytes migrate upstream after completing the leukocyte adhesion cascade

**DOI:** 10.1080/19336918.2019.1587269

**Published:** 2019-03-17

**Authors:** Nicholas R. Anderson, Alexander Buffone, Daniel A. Hammer

**Affiliations:** a Department of Chemical and Biomolecular Engineering, University of Pennsylvania, Philadelphia, PA, USA; b Department of Bioengineering, University of Pennsylvania, Philadelphia, PA, USA

**Keywords:** P-selectin, ICAM-1, SDF-1α, CXCL12, Flow chamber, Directional migration

## Abstract

The leukocyte adhesion cascade is of critical importance for both the maintenance of immune homeostasis and the ability of immune cells to perform effector functions. Here, we present data showing CD4^+^ T cells migrate upstream (against the direction of flow) after completing the leukocyte adhesion cascade on surfaces displaying either ICAM-1 or ICAM-1 and VCAM-1, but migrate downstream on surfaces displaying only VCAM-1. Cells completing the cascade on HUVECs initially migrate upstream before reverting to more random migration, partly caused by transmigration of cells migrating against the flow. Furthermore, cells migrating upstream transmigrate faster than cells migrating downstream. On HUVECs, blocking interactions between LFA-1 and ICAM-1 resulted in downstream migration and slower transmigration. These results further suggest a possible physiological role for upstream migration *in vivo*.

## Letter text

All immune cells, including T cells, need to be able to traffic between the lumen of the vasculature and tissues to maintain immune homeostasis or to perform effector functions at the sites of infection and inflammation [1]. In order to perform these duties, immune cells need to adhere to the endothelium near sites of activation while avoiding sites where no response is needed. In response to these challenges, nature has evolved the leukocyte adhesion cascade, which is typically separated into four distinct, sequential steps: tethering, rolling, firm arrest, and transmigration [2]. During tethering, cells interact with P- or E-selectin molecules expressed on the activated endothelial surface. These initial interactions slow the cell while also allowing more intimate contact between the cell and the apical surface of endothelium [2,3]. As the cell velocity slows, additional selectins and other adhesive molecules are engaged, leading to sustained or slow rolling. During rolling, the T lymphocyte can be activated by chemokines, either on the endothelium or in solution, which initiates intracellular signalling cascades whose end result is the activation of integrins such as LFA-1 on the T cell surface. Once fully activated, the integrin-based adhesions become mechanically stronger and lead to firm arrest on the endothelial surface [2,4,5]. Along these lines, we recently experimentally recreated the leukocyte adhesion cascade *in vitro* and identified the requirements for rolling and firm adhesion in the form of a concise state diagram [6]. After adhesion, T cells spread and migrate along the apical surface of endothelium to find an appropriate location to transmigrate across the endothelium [2,7].

Previously, it has been shown that lymphocytes will crawl upstream against the direction of flow on surfaces coated with ICAM-1. On surfaces containing ICAM-1, T and marginal zone B cells, along with haematopoietic stem and progenitor cells (HSPCs), will migrate against the direction of flow – that is, upstream. On surfaces displaying VCAM-1, these same cells migrate with the direction of flow [8–11]. In the case of T cells, small amounts of ICAM-1 on a surface containing mostly VCAM-1 provide enough stimulus for directed migration upstream [9]. However, these migration studies were conducted on surfaces displaying purified proteins where cells were allowed to settle prior to the initiation of shear flow. Thus, it is unknown whether the multiple factors that affect chemokine-mediated arrest or selectin-mediated tethering and rolling would interfere with upstream migration. Previous *in vivo* studies in rats showed that T-cells indicated a preference for upstream migration in meningeal CNS structures [12]. Separately, it has been shown that LFA-1-I/CAM-1 interactions are critical for mediating upstream migration on a model of the murine brain microvasculature, and showed that only ICAM-1 can support upstream migration while ICAM-2 only allows cells to migrate perpendicular to the direction of flow [13]. These studies suggest that upstream migration is physiologically relevant and observable on endothelium. Here, we show that primary human CD4^+^ T cells migrate upstream after completing the leukocyte adhesion cascade on surfaces containing concentrations of purified adhesion molecules and chemokines required for T-cell arrest, as well as on stimulated endothelium, thus developing a tool that allows us to understand the entire progression from tethering to arrest to upstream migration quantitatively.

**Figure 1.**
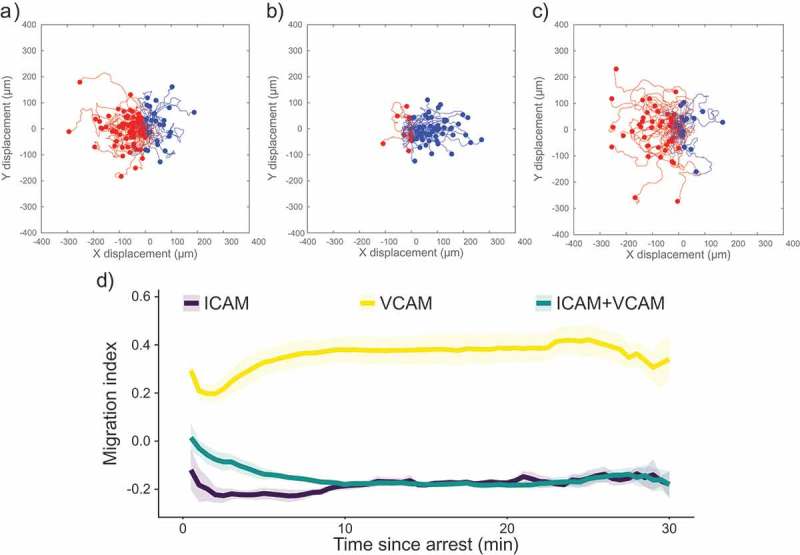
Scattergrams of cell tracks of cells migrating on surfaces containing (a) ICAM-1, (b) VCAM-1, or (c) ICAM-1 and VCAM-1. Tracks have been adjusted to all start at the same point. Red tracks indicate cells migrating against the direction of flow and blue tracks indicate cells migrating with the direction of the flow. All images have flow from left to right at 100 s^−1^. (d) Plot of migration index over time for surfaces coated with recombinant proteins. Upstream migration is indicated by a negative migration index, downstream migration by positive values, and random migration by values near zero. ICAM-1 surfaces support upstream migration while VCAM-1 surfaces do not. Surfaces containing ICAM-1 and VCAM-1 support upstream migration to a similar extent as ICAM-1-only surfaces. Data presented as mean ± SEM, n = 4 independent experiments.

Figure 1 shows scattergrams of paths taken by migrating CD4^+^ T cells after arrest on surfaces containing ICAM-1 only, VCAM-1 only, and ICAM-1 and VCAM-1 (Figure 1(a–c)). These surfaces, and all recombinant protein surfaces described in this paper, displayed P-selectin to support recruitment from the free stream, along with SDF-1α (CXCL12) to cause integrin activation. Experiments were performed at a shear rate of 100 s^−1^. For simplicity, we will refer to surfaces based solely on the integrin ligand presented (ICAM-1, VCAM-1, or both). We also characterized the T-cells using flow cytometry, finding that they were >98% CD3^+^CD4^+^ (Supplemental Figure 1). In Figure 1, upstream trajectories are coloured red, and downstream trajectories are coloured blue. After the arrest, the majority of cells migrate upstream (against the direction of flow) on surfaces containing ICAM-1, as highlighted by the preponderance of red tracks in Figure 1. In contrast, surfaces containing only recombinant VCAM-1 did not support upstream migration, as shown by the preponderance of blue tracks. Surfaces containing both recombinant ICAM-1 and VCAM-1 show a similar ratio of cells migrating upstream as surfaces containing only ICAM-1, consistent with a previous report from our laboratory that small amounts of ICAM-1 support upstream migration [9]. We quantified the direction of migration using the Migration Index (MI) in Figure 1(d). The MI is the ratio of motion against the direction of flow to the total distance migrated by the cell, with negative values indicating upstream migration. On surfaces displaying only ICAM-1 or only VCAM-1, the migration index does not change significantly over the time course of the experiment. Although some cells initially migrate downstream on ICAM-1 surfaces, cells often begin to migrate upstream within the first 30 s of their migrating. On surfaces containing only VCAM-1, cells showed largely downstream migration, as indicated by the positive MI. At long times, surfaces containing both ICAM-1 and VCAM-1 did not show a difference in MI compared to ICAM-1-only surfaces. However, on surfaces containing both ICAM-1 and VCAM-1 cells took longer to reach their steady state response. In summary, these results show that the presence of chemokine and selectin do not adversely affect the ability of CD4^+^ T cells to migrate against the direction of flow in a manner dependent on LFA-1/ICAM-1 interactions.

**Figure 2.**
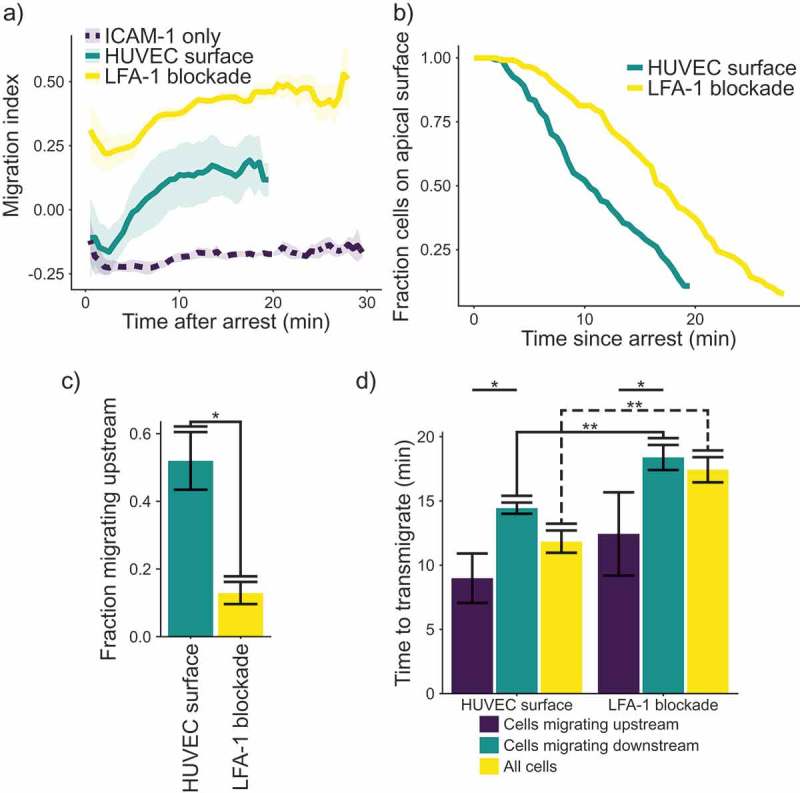
(a) Plot of migration index over time. Upstream migration is indicated by a negative migration index, downstream migration by positive values, and random migration by values near zero. Blockade of LFA-1 prevents upstream migration on TNFα stimulated HUVECS, while cells with unblocked LFA-1 initially migrate upstream before reverting to downstream migration. ICAM-1-only recombinant protein surface data is provided for comparison. Data presented is mean ± SEM, n = 4 independent experiments. (b) Plot showing the remaining fraction of tracked cells at each time point. Cells on HUVEC monolayers were tracked from initial migration to transmigration or the end of the experiment, whichever is sooner. (c) Comparison of fraction of cells which migrated upstream on HUVEC monolayers with or without LFA-1 blockade. Data presented is mean ± SEM, n = 4 independent experiments. (d) Comparison of the time from arrest to transmigration on HUVEC monolayers with or without LFA-1 blockade. Data presented is mean ± SEM, n = 4 independent experiments. * p < 0.05, ** p < 0.005.

In order to connect the observation of upstream migration to physiology, we measured the migration of cells on stimulated HUVECs after rolling and arrest (Figure 2). HUVEC monolayers were activated using 200 ng mL^−1^ TNF-α and used 4 h after stimulation to ensure interaction with the T cells. Cells interacting with a HUVEC monolayer show upstream migration, similar to T cells migrating on a surface consisting of ICAM-1-only or ICAM-1 and VCAM-1 (plus selectin and chemokine) (Figure 2(a)). However, on the endothelial surfaces, although cells initially crawl upstream, upstream migration is not sustained, as T cells begin to migrate randomly after an initial upstream “burst.“ This result is in comparison to the results on molecularly coated surfaces, which show a consistent MI for the entire experiment. Also, we found that blocking LFA-1-ICAM-1 interactions with an antibody abolished any migration against the fluid flow, once again highlighting the importance of this interaction for upstream migration. We surmised that one possible cause of the loss of upstream migration on endothelium might be the transmigration of cells across the endothelial layer. Since we stopped tracking cells once they had transmigrated, changes in the distribution of cells we track might explain the increase in the migration index. We hypothesized that the change in MI over time might be because the most potent upstream migrators are lost to transmigration, skewing the distribution of directionally migrating cells that remain. Indeed, we found that cells treated with an LFA-1-blocking antibody remain on the apical surface of the HUVEC monolayer longer than untreated cells (Figure 2(b)). Treatment with the LFA-1 blocking antibody also reduces the fraction of cells migrating upstream (Figure 2(c)). We then measured the time from arrest to transmigration, with and without an antibody against LFA-1 (Figure 2(d)). On stimulated HUVECs, there was a significant difference in the time to transmigrate between cells migrating upstream and those migrating downstream. Also, blocking LFA-1 increases the time required for transmigration. In addition, blocking LFA-1 significantly increases the time to transmigrate for cells migrating downstream, as well as the overall time to transmigrate for all cells. Interestingly, the period for transmigration almost exactly coincides with the time delay for the decay of directional migration to random migration. We also tested the cells on HUVECs which had been stimulated with TNFα for 48 h to see if changing ratios of ICAM-1 and VCAM-1 would influence the migration, but we did not see any change in our metrics of transendothelial migration (Supplemental Figure 2).

To our knowledge, this is the first report of primary human T cells showing upstream migration after completing the entire leukocyte adhesion cascade, and the first demonstration that CD4^+^ T-cells crawl upstream after the imposition of shear flow on endothelium. T cells choose a direction of migration within 30 s of arresting on surfaces containing P-selectin and chemokine, and either ICAM-1, VCAM-1, ICAM-1 and VCAM-1, or a monolayer of HUVECs. This is in line with previous results showing the T cells migrate upstream in the presence of even minimal amounts of ICAM-1 [9]. In addition, a previous study has suggested the importance of the uropod in determining the direction of migration for T cells under flow [14]. However, our CD4^+^ T cells on surfaces containing only ICAM-1 started to migrate upstream before an obvious morphological polarity was established, suggesting that there may be additional regulatory mechanisms controlling this behaviour. In contrast, cells on surfaces containing only VCAM-1 began migrating downstream before polarity was established. Surfaces containing both ICAM-1 and VCAM-1 allowed enough time for cells to establish morphological polarity before reaching a maximal response. We also showed that T cells migrating on HUVECs initially prefer to migrate upstream, before reverting to more random migration. This reversion is driven by cells migrating upstream crossing the endothelial layer faster than cells migrating with the direction of flow. Blocking LFA-1 interactions on a stimulated HUVEC surface resulted in consistent downstream migration, as well as significantly longer time to transmigrate, in line with previous reports suggesting a large role for LFA-1 stimulation in transmigration [15]. In summary, these results suggest that migration against the direction of flow in CD4^+^ T cells is a robust response to their environment and possibly has a physiologically important function in vivo by directing T cells to their recruitment sites faster.

### Adsorption of protein A/G and SDF-1α

Non-tissue culture treated polystyrene Petri dishes (Corning, Cat. 430588) were enclosed using a single well FlexiPerm gasket (Sarstedt, Cat. 94–6032-039). A solution of 2 µg mL^−1^ of Protein A/G (Thermo-Fisher Scientific, Cat. PI21186) and 1 µg mL^−1^ of SDF-1α (R&D Systems, Cat. 350-NS) was applied and incubated overnight at 4°C. The surfaces were then washed three times with PBS before a 30-min incubation of a 0.2% w/v solution of Pluronic F-127 (Sigma-Aldrich, Cat. P2443). The surfaces were washed again three times with PBS. Next, a solution of ICAM-1/Fc and/or VCAM-1/Fc and P-selectin/Fc chimera (R&D Systems, Cat. 720-IC, 862-VC, 137-PS, respectively) was applied to the surface and incubated for 3 h at room temperature. The completed surfaces were then washed again three times with PBS before use.

### CD4^±^ T cells

Purified primary human CD4^+^ T cells from anonymized donors were obtained from the University of Pennsylvania Human Immunology Core. Cells were resuspended in RPMI-1640 media (Gibco, Cat. 724000) supplemented with 10% FBS (Sigma-Aldrich, Cat. F2442) and used immediately.

### HUVECs

Human Umbilical Vein Endothelial Cells (HUVECs) were maintained in EBM-2 growth media with BulletKit supplement (Lonza, Cat. CC-3162). For experimentation, HUVEC were seeded in a 35 mm x 10 mm TC treated dish (Corning, Cat. 353001) and grown to confluence. HUVECs were then stimulated with 200 ng mL^−1^ TNFα (BioLegend, Cat. 570106) for 4 h or 48 h prior to experimentation.

### LFA-1 blocking

Cells were resuspended at a concentration of 1 × 10^7^ mL^−1^. Cells were blocked using a functional blocking anti-CD11a antibody (Clone HI111, BioLegend, Cat. 301214) at a final concentration of 50 µg mL^−1^ for 30 min at 37°C and 5% CO_2_. Cells were then diluted to 5 × 10^5^ mL^−1^ and used immediately.

### Flow cytometry

Immunofluorescence staining and flow cytometric analysis of cells were performed as described previously [10]. Cells were washed twice in PBS and resuspended at 1 × 10^6^ mL^−1^. Samples were incubated with combinations of fluorescently labelled antibodies MOPC-21-PE (IgG1κ control, Cat. 400111), MOPC-21-FITC (IgG1κ control, Cat. 400107), MOPC-21-APC (IgG1κ control, Cat. 400119), UCHT1-APC (anti-CD3, Cat. 300412), RPA-T4-PE (anti-CD4, Cat. 300508), JS-83-FITC (anti-CD45RA, eBioscience, Cat. 11–9979-41), KPL-1-PE (anti-PSGL-1, Cat. 328805), and V S056-PE (anti-CD62L, Cat. 304805). All antibodies were from BioLegend unless otherwise noted. Flow cytometric analysis was performed on a Accuri C6 using the Accuri C6 Analysis software (BD Biosciences, San Jose, CA). Histograms were generated using the FlowJo software package (FlowJo, Ashland, OR).

### Flow chamber assays

Flow chamber experiments were performed in a circular parallel plate flow chamber (GlycoTech, Gaithersburg, MD) with a gasket width of 0.25 cm, thickness of 127 µm, and length of 2 cm. Before flow chamber experiments, a functionalized dish was washed with prewarmed PBS and media to remove air bubbles in the flow path. The assembled flow chamber was mounted on an inverted Axiovert 200 (Carl Zeiss, Gottigen, Germany) enclosed by a XL-3 microscope incubator (PeCon, Ulm, Germany). All experiments were performed at 37°C. T cells were suspended at a concentration of 5 × 10^5^ mL^−1^ and were perfused into the flow chamber via syringe pump (Harvard Apparatus, Holliston, MA) at a flow rate corresponding to a calculated wall shear rate of 100 s^−1^. Rolling and adhesion of T cells on the immobilized adhesive ligands was observed via phase contrast microscopy under a 10X objective (NA = 0.2, Type A-Plan, Carl Zeiss). All recombinant protein surfaces were tested in triplicate on four different days with different anonymized donors and the results averaged. HUVEC surfaces were tested in duplicate on four different days with different anonymized donors.

### Data acquisition and cell tracking

A CCD camera (QImaging, Surrey, BC, Canada) was used to monitor T cell adhesion events with adhesive substrates. Adhesion of T cells was recorded on DVD+RW discs for cell tracking analyses. Cell adhesion videos were redigitized to 6400×480 pixels at 29.97 frames s^−1^ and deinterlaced with HandBrake software (http://handbrake.fr/) then converted to image stacks with MATLAB (MathWorks, Natick, MA). Images for every 30 s were obtained and migrating cells were manually tracked using the MTrackJ plugin (https://imagescience.org/meijering/software/mtrackj/) in the ImageJ program (http://imagej.nih.gov/ij; National Institutes of Health). Cells on surfaces of recombinant proteins were tracked from the time of their spreading to the end of the experiment or when the cell stopped migrating, whichever was sooner. Cells on HUVEC monolayers were tracked from initial spreading to transmigration, as indicated by phase brightness change. These points were then analysed using a custom MATLAB script to determine the migration index of each cell. Statistical tests were performed using a paired Student’s t-test.
