# Supplementary material for: T lymphocytes migrate upstream after completing the leukocyte adhesion cascade
Source: Cell Adh Migr. 2019 Mar 17;13(1):163–8. doi: 10.1080/19336918.2019.1587269 (PMC6527389; doi:10.1080/19336918.2019.1587269)
Supplement: Supplemental Material [file kcam-13-01-1587269-s001.zip › Supplemental Materials Final.docx]

Supplemental Figures for Anderson, Buffone, and Hammer, “**T lymphocytes migrate upstream after completing the leukocyte adhesion cascade**”


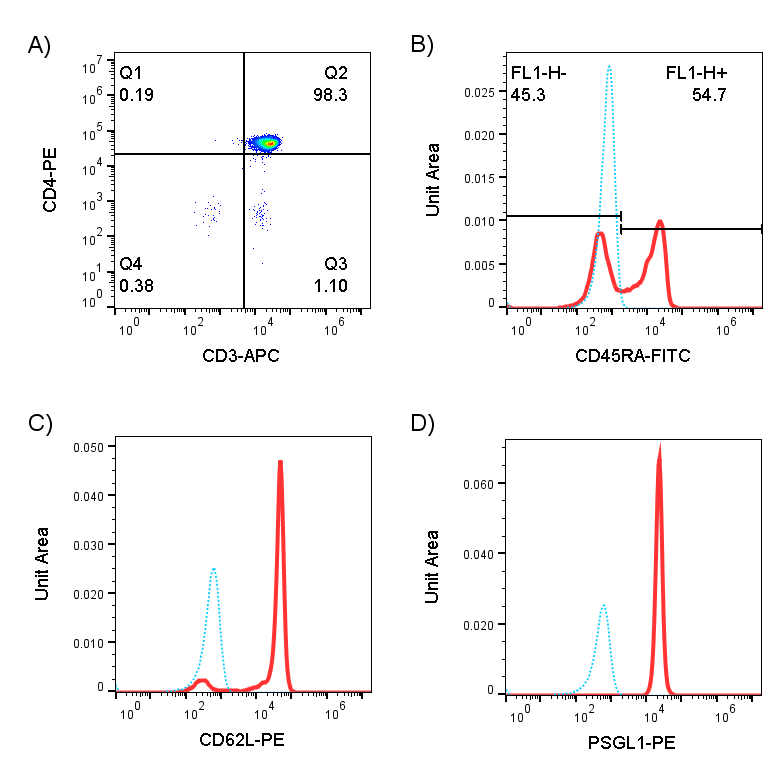


**Supplemental Figure 1:**

Flow cytometric characterization of primary human T cells. (A) Cells are > 98% CD3+CD4+ T cells by flow cytometry. (B) Roughly 55% of cells are CD45RA+, indicating that slightly over half of cells are naïve T cells. (C) Cells robustly express L-selectin (CD62L) and (D) PSGL-1, indicating their ability to perform the leukocyte adhesion cascade.


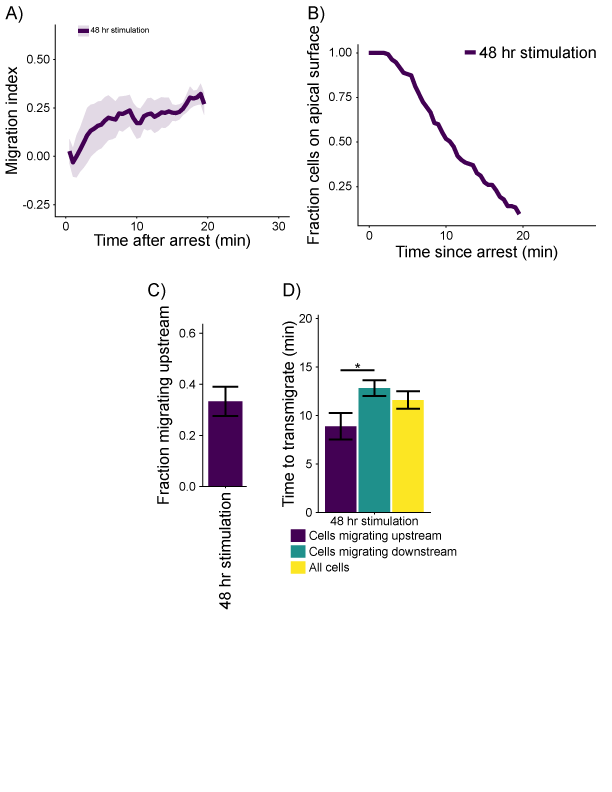


**Supplemental Figure 2:**

(A) Plot of migration index over time for surfaces of HUVECs stimulated for 48 hours with TNFα. Upstream migration is indicated by a negative migration index, downstream migration by positive values, and random migration by values near zero. Data presented is mean ± SEM, n = 4 independent experiments. (B) Plot showing the remaining fraction of tracked cells at each time point. Cells on HUVEC monolayers were tracked from initial migration to transmigration or the end of the experiment, whichever is sooner. (C) Comparison of fraction of cell which migrated upstream. Data presented is mean ± SEM, n = 4 independent experiments. (D) Comparison of the time from arrest to transmigration. Data presented is mean ± SEM, n = 4 independent experiments. * p < 0.05.
